# Supplementary material for: Genetic accommodation via modified endocrine signalling explains phenotypic divergence among spadefoot toad species
Source: Nat Commun. 2017 Oct 19;8:993. doi: 10.1038/s41467-017-00996-5 (PMC5648835; doi:10.1038/s41467-017-00996-5)
Supplement: Supplementary file 1 — Supplementary Information [file 41467_2017_996_MOESM1_ESM.pdf]

Supplementary Table 1. Statistical results from experiments testing the responsiveness of three spadefoot toad species to pond drying.

Model term	df	-2ΔLogLik	*P*
(A) Time to metamorphosis			
Species	2	163.73	<0.0001
Water level	1	32.76	<0.0001
Species x Water level	2	49.76	<0.0001
(B) Thyroid hormone – Differences across stages			
Species	1	14.67	0.0001
Stages	1	25.90	<0.0001
Species x Treatment	1	1.29	0.255
(C) Corticosterone – Differences across stages			
Species	2	10.5	0.005
Stages	1	8.90	0.003
Species x Treatment	2	2.91	0.234
(D) Metabolic rate - Differences across stages			
Species	2	88.37	<0.0001
Stages	2	10.83	0.004
Species x Treatment	4	22.38	0.0002
(E) Thyroid hormone – Response to water drop			

Species	1	11.98	0.0005
Water level	1	6.09	0.014
Species x Water level	1	11.68	0.0006

(F) Corticosterone – Response to water drop

Species	2	25.24	<0.0001
Water level	1	15.73	<0.0001
Species x Water level	2	11.04	0.004

(G) Metabolic rate – Response to water drop and hormonal manipulation

Species	2	158.48	<0.0001
Treatment	3	14.67	0.002
Species x Treatment	6	11.67	0.069

(A) We found differences in the timing of metamorphosis depending on the water level, but not all species showed the same degree of responsiveness. (B, C) Species showed constitutive, stage-matched differences in thyroid hormone and corticosterone levels. (D) Metabolic rate also differed among species and developmental stages. (E, F) Thyroid hormone and corticosterone increased in response to pond drying, but species differed in the extent of this endocrine regulation. (G) Metabolic rate varied across species and in response to addition of exogenous corticosterone or reduced water level, but not all species were equally responsive.
